# Transient Ingrowth of Lymphatic Vessels into the Physiologically Avascular Cornea Regulates Corneal Edema and Transparency

**DOI:** 10.1038/s41598-017-07806-4

**Published:** 2017-08-03

**Authors:** Deniz Hos, Anne Bukowiecki, Jens Horstmann, Felix Bock, Franziska Bucher, Ludwig M. Heindl, Sebastian Siebelmann, Philipp Steven, Reza Dana, Sabine A. Eming, Claus Cursiefen

**Affiliations:** 1 Department of Ophthalmology, University of Cologne, Köln, Germany; 2 Center for Molecular Medicine Cologne (CMMC), University of Cologne, Köln, Germany; 3 Excellence Cluster: Cellular Stress Responses in Aging-associated Diseases, CECAD, University of Cologne, Köln, Germany; 4 Schepens Eye Research Institute, Boston, MA USA; 5 Department of Dermatology, University of Cologne, Köln, Germany

## Abstract

Lymphangiogenesis is essential for fluid homeostasis in vascularized tissues. In the normally avascular cornea, however, pathological lymphangiogenesis mediates diseases like corneal transplant rejection, dry eye disease, and allergy. So far, a physiological role for lymphangiogenesis in a primarily avascular site such as the cornea has not been described. Using a mouse model of perforating corneal injury that causes acute and severe fluid accumulation in the cornea, we show that lymphatics transiently and selectively invade the cornea and regulate the resolution of corneal edema. Pharmacological blockade of lymphangiogenesis via VEGFR-3 inhibition results in increased corneal thickness due to delayed drainage of corneal edema and a trend towards prolonged corneal opacification. Notably, lymphatics are also detectable in the cornea of a patient with acute edema due to spontaneous Descemet´s (basement) membrane rupture in keratoconus, mimicking this animal model and highlighting the clinical relevance of lymphangiogenesis in corneal fluid homeostasis. Together, our findings provide evidence that lymphangiogenesis plays an unexpectedly beneficial role in the regulation of corneal edema and transparency. This might open new treatment options in blinding diseases associated with corneal edema and transparency loss. Furthermore, we demonstrate for the first time that physiological lymphangiogenesis also occurs in primarily avascular sites.

## Introduction

The lymphatic vascular system is essential for fluid and lipid homeostasis and is critically involved in the regulation of inflammation, immunity, and cancer dissemination^1, 2^. Therefore, lymphatic vessels are present in almost every tissue of the organism. One exception is the transparent cornea, which is physiologically devoid of both blood and lymphatic vessels^3^. This state, which is termed corneal “angiogenic privilege”, is essential for proper vision and is actively maintained by several antiangiogenic mechanisms^4–10^. However, this angiogenic privilege of the cornea is not invulnerable: whereas minor inflammatory vascular stimuli are buffered and do not induce an angiogenic response, severe tissue damage and inflammation can “overwhelm” the cornea’s antiangiogenic mechanisms and lead to a secondary ingrowth of blood and clinically invisible lymphatic vessels from the adjacent limbus towards the central cornea^3, 11^. Studies have demonstrated the presence of corneal lymphatic vessels in various injury models including chemical injury such as silver nitrate^12^ or alkali burn^13^, thermal injury^14^, corneal suture placement^15^, herpetic keratitis^16^, and cornea transplantation^17^. In these models, lymphatic vessels and blood vessels seem to grow in parallel into the cornea. In contrast, corneal lymphangiogenesis without concurrent hemangiogenesis has been detected in mouse models of dry eye and allergic disease^18, 19^. Although the knowledge on the specific function of corneal lymphatic vessels is growing, a functional role for these vessels has only been demonstrated in the context of corneal transplantation, dry eye and allergic disease^20, 21^. In these diseases, lymphatic vessels seem to facilitate the migration of antigen presenting cells from the cornea to the regional lymph nodes, where accelerated sensitization against allo- or autoantigens occurs^5, 18, 19, 21–24^. Thus, corneal lymphatic vessels seem to contribute to the induction and progression of corneal transplant rejection, dry eye and allergic disease and are therefore mostly considered undesirable. Importantly, blockade of corneal lymphangiogenesis has been shown to promote corneal transplant survival and ameliorate dry eye or allergic disease^17, 19, 22, 23, 25^.

Although a plethora of studies have demonstrated pathological and undesirable functions for corneal lymphangiogenesis, evidence for beneficial, physiological functions for corneal lymphangiogenesis in clinically relevant diseases was so far missing. In contrast, studies in extraocular tissues, such as the skin, demonstrate that lymphangiogenesis is important e.g. for appropriate wound repair. In this regard, lymphatic vessels regulate tissue pressure, prevent the development of edema and allow the drainage and removal of fluid and cell debris from the inflamed site^1, 2, 26^. Moreover, lymphangiogenesis is also important to terminate inflammatory responses in the skin, as recent studies indicate that the blockade of cutaneous lymphatic vessels can result in chronic inflammation and edema, whereas the specific activation of lymphatic vessels can ameliorate these conditions^27–29^.

It remains elusive, whether lymphatic vessels at the cornea might also have physiological, possibly beneficial functions after tissue damage, e.g. facilitating adequate wound healing or regulation of corneal edema. We therefore investigated whether a corneal injury that causes acute corneal edema is accompanied by the ingrowth of lymphatic vessels and whether corneal lymphangiogenesis contributes to the physiological healing response.

## Results

### Corneal perforating incision injury results in transient corneal edema and selective corneal lymphangiogenesis

**Figure 1:**
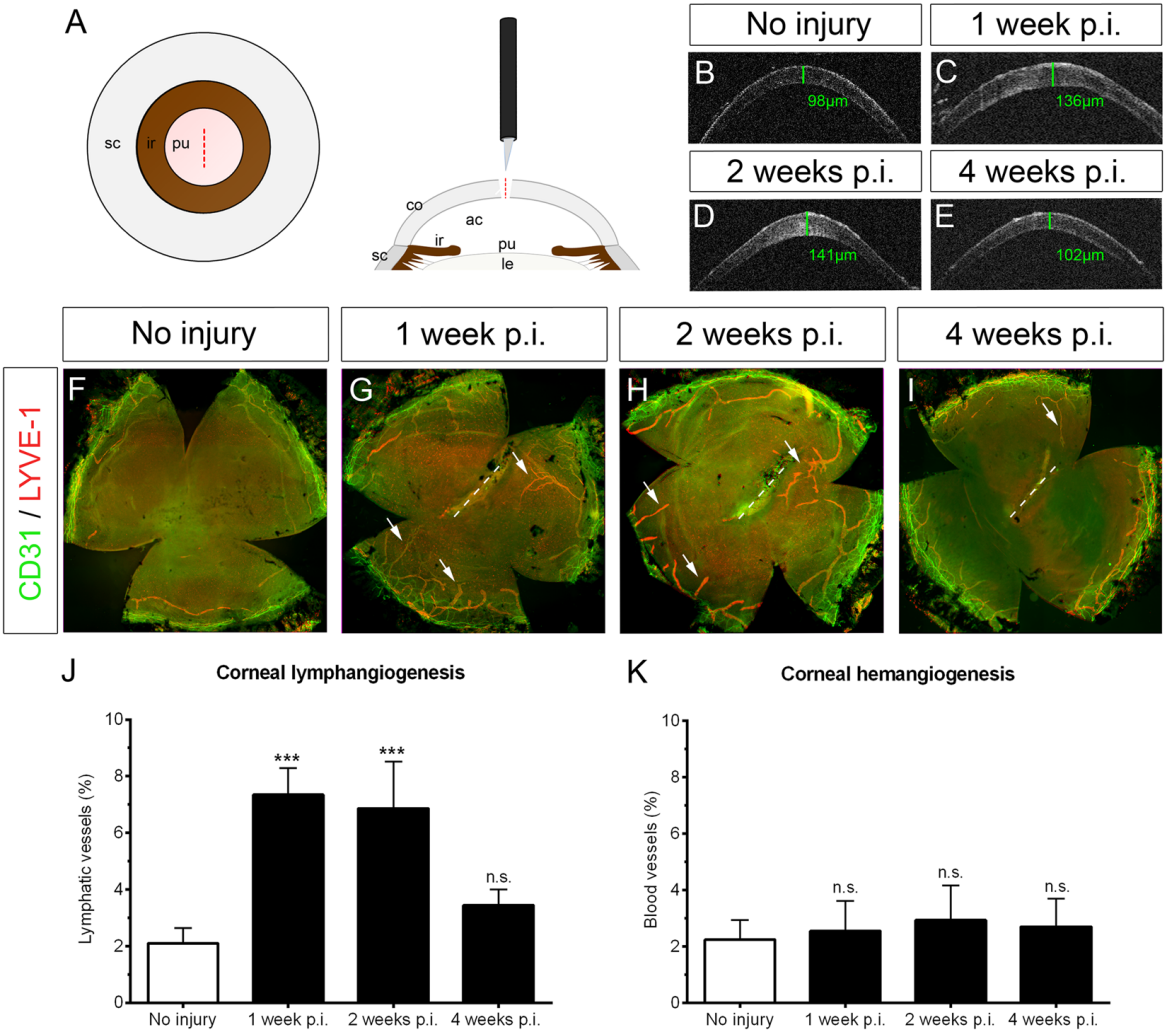
Corneal perforating incision injury induces transient edema and results in isolated lymphangiogenesis without hemangiogenesis. (**A**) Mouse model of central perforating corneal incision injury. After application of atropine to dilate the pupil and avoid iris incarceration, a linear perforating corneal incision (1.0 mm length) is performed (dashed lines). The lens and iris are not touched. Ac: anterior chamber; co: cornea; ir: iris; le: lens; pu: pupil; sc: sclera. (**B**–**E**) *In vivo* optical coherence tomography scans of the anterior segment demonstrate a transient increase of corneal thickness as a measure of corneal edema; green bars: areas of corneal thickness measurements; green values: central corneal thickness. (**F**–**I**) Corneal whole mounts stained with LYVE-1 (red) and CD31 (green); dashed lines: area of incision injury. LYVE-1^high^/CD31^low^ lymphatic vessels (arrows) growing towards the central cornea are detectable 1 week after injury and persist until 2 weeks after injury. Afterwards, lymphatic vessels regress and the area covered by lymphatic vessels after 4 weeks is comparable to uninjured corneas. In contrast to lymphatic vessels, no significant ingrowth of LYVE-1^neg^/CD31^high^ blood vessels into the cornea is detectable; p.i.: post injury. (**J–K**) Quantification of corneal lymph- and hemangiogenesis; data are shown as mean + SD; n = 5–10 per time point; statistical analysis was performed using the unpaired 2-tailed Student’s t test: ***: p < 0.001; n.s.: not significant.

We first analyzed whether a corneal injury that leads to a profound increase in corneal edema results in corneal lymphangiogenesis. For this purpose, we used an established mouse model of perforating corneal incision injury, which is known to induce transient edema formation and opacification^30^ (Fig. 1A to E). Immunohistochemical analysis of corneal whole mounts at various time points after injury revealed that corneal incision injury results in significant corneal lymph- but not hemangiogenesis. LYVE-1^high^/CD31^low^ lymphatic vessels growing towards the central cornea were clearly detectable 1 week after injury (lymphvascularized area in uninjured: 2.09% versus after 1 week: 7.35%, p < 0.001; Fig. 1F,G) and persisted until 2 weeks after injury (after 2 weeks: 6.87%, p < 0.001; Fig. 1H). Afterwards, corneal lymphatic vessels regressed. After 4 weeks, the amount of corneal lymphangiogenesis was comparable to uninjured corneas (after 4 weeks: 3.45%, p > 0.05; Fig. 1I). In contrast to corneal lymphatic vessels, we could not detect any ingrowth of LYVE-1^neg^/CD31^high^ blood vessels into the cornea after injury (Fig. 1F to I). In addition, we analyzed gene expression of the major prolymphangiogenic growth factors VEGF-C and VEGF-D and the corresponding receptor VEGFR-3 by real-time PCR. Incision injury led to a significant increase in corneal VEGF-C and VEGF-D expression: VEGF-C and VEGF-D mRNA expression levels peaked at 1 week after injury and declined afterwards (Supplemental Figure 1A and B). Similarly, VEGFR-3 mRNA expression was also strongly upregulated: expression levels were significantly increased after 1 week and decreased afterwards, but were still elevated when compared to uninjured corneas (Supplemental Figure 1C). Gene expression of VEGFR-2, which can in addition to VEGF-C also be activated by VEGF-A and induce corneal hemangiogenesis, was significantly reduced during the first 2 weeks after injury before returning to normal levels after 4 weeks (Supplemental Figure 1D). We also analyzed mRNA expression of the soluble forms of VEGFR-2 and VEGFR-3 (sVEGFR-2 and sVEGFR-3, respectively), which both have been shown to contribute to corneal alymphaticity^5, 6^. After incision injury, mRNA expression of sVEGFR-2 significantly decreased after 1 and 2 weeks and returned to normal values after 4 weeks (Supplemental Figure 1E), whereas mRNA expression of sVEGFR-3 was upregulated at later time points (Supplemental Figure 1F). Gene expression of PROX-1, a master regulator of lymphatic endothelial cell differentiation, was significantly elevated at all analyzed time points (Supplemental Figure 1G).

Thus, an incisional perforating corneal injury results in transient corneal edema and the transient and selective ingrowth of lymphatic, but not blood vessels into the cornea.

### Blockade of corneal lymphangiogenesis results in delayed resolution of corneal edema

**Figure 2:**
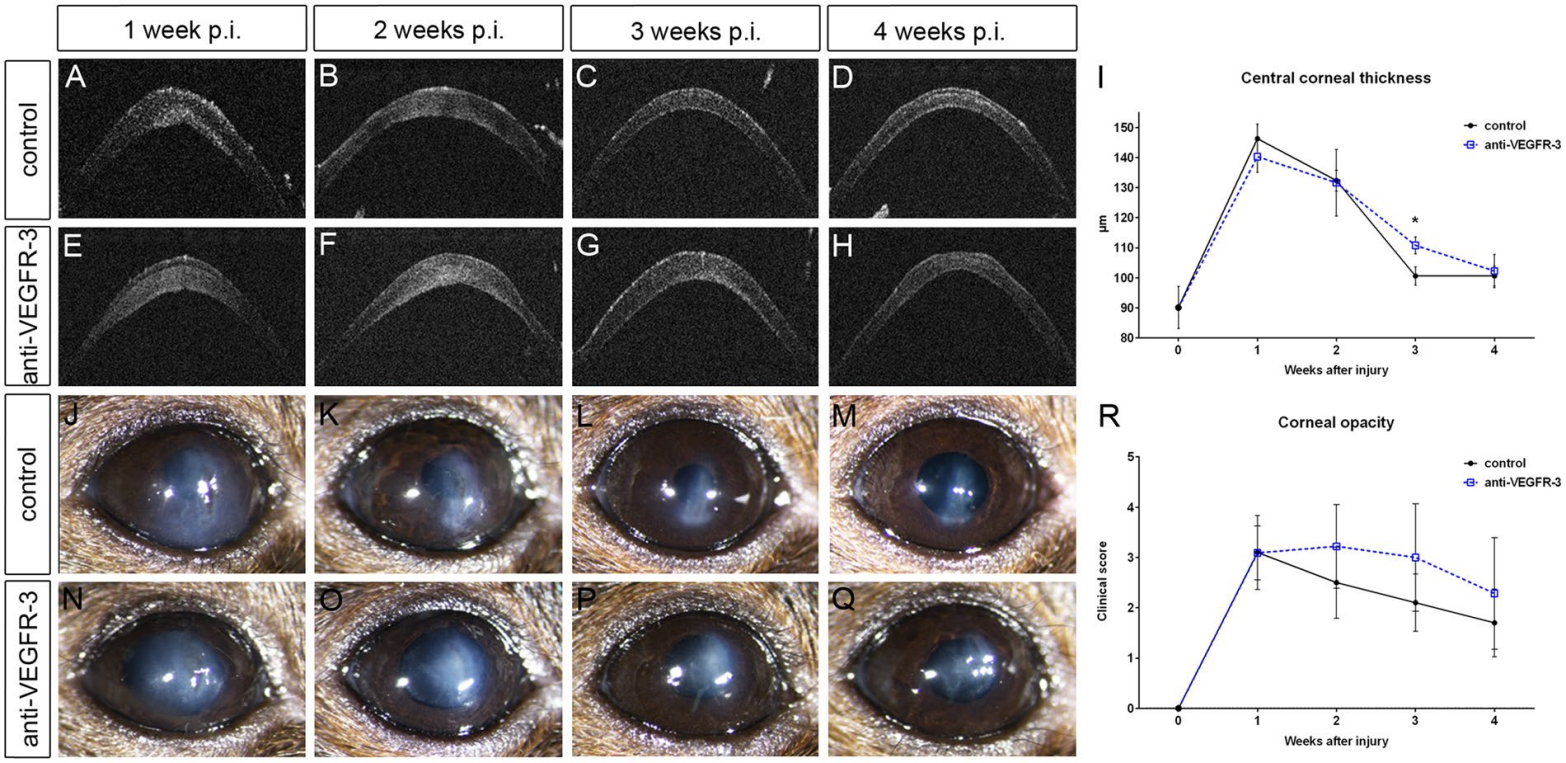
Blockade of lymphangiogenesis results in delayed resolution of corneal edema and prolonged opacification. Mice were treated with the anti-VEGFR-3 antibody mF4-31C1 (500 µg, intraperitoneally) directly before, on day 3, 6, 9, and 12 after incision injury or equal amounts of PBS. **(A**–**H)** *In vivo* optical coherence tomography scans of the anterior segment measuring central corneal thickness (CCT); **(I)** Blockade of corneal lymphangiogenesis resulted in delayed regression of CCT. (**J**–**Q**) Clinical images of injured corneas; **(R)** Blockade of lymphangiogenesis resulted in a trend towards higher opacity scores. p.i.: post injury; data are shown as mean + /− SD; n = 5–10 per group per time point; statistical analysis was performed using the unpaired 2-tailed Student’s t test *: p < 0.05.

In extraocular tissues, lymphatic vessels are known to mediate fluid homeostasis and clearance of cells and debris after tissue damage^1, 26^. We hypothesized that lymphangiogenesis might also regulate corneal edema and therefore determine whether blockade of corneal lymphangiogenesis after incision injury has an impact on corneal edema and transparency. For this purpose, we treated mice with the anti-VEGFR-3 antibody mF4-31C1 after injury. Treatment with mF4-31C1 resulted in a significant inhibition of corneal lymphangiogenesis (lymphvascularized area after 1 week: controls: 8.87% versus mF4-31C1: 4.86%, p < 0.001; after 2 weeks: controls: 10.88% versus mF4-31C1: 4.93%, p < 0.001; Supplemental Figure 2). *In vivo* optical coherence tomography (OCT) scans of the anterior segment showed that injured corneas developed significant corneal edema noticeable in an increase of central corneal thickness (CCT). CCT peaked within the first 2 weeks and then gradually regressed. Importantly, blockade of lymphangiogenesis resulted in delayed regression of CCT (CCT in uninjured: 90.1 µm; after 1 week: controls: 146.3 µm versus mF4-31C1: 140.3 µm, p > 0.05; after 2 weeks: controls: 132.4 µm versus mF4-31C1: 131.7 µm, p > 0.05; after 3 weeks: controls: 100.6 µm versus mF4-31C1: 110.8 µm, p < 0.05; after 4 weeks: controls: 101.4 µm versus mF4-31C1: 102.3 µm, p < 0.05; Fig. 2A to I). In addition, we clinically scored corneal opacity after incision injury in a blinded fashion. Similar to CCT, corneal opacity peaked in the first 2 weeks and then gradually decreased until corneas became almost entirely clear after 4 weeks. Blockade of lymphangiogenesis by mF4-31C1 resulted in a trend towards higher opacity scores, although the differences did not reach statistical significance (opacity score in uninjured: 0; after 1 week: controls: 3.1 versus mF4-31C1: 3.1; after 2 weeks: controls: 2.5 versus mF4-31C1: 3.2; after 3 weeks: controls: 2.1 versus mF4-31C1: 3.0; after 4 weeks: controls: 1.7 versus mF4-31C1: 2.5; all p-values > 0.05; Fig. 2J to R). Therefore, our experiments show that blockade of corneal lymphangiogenesis after incision injury leads to an increase of CCT - arguably due to a delay in the resolution of corneal edema - and a trend towards prolonged opacification.

### Acute edema in a patient with keratoconus is associated with selective corneal lymphangiogenesis

**Figure 3:**
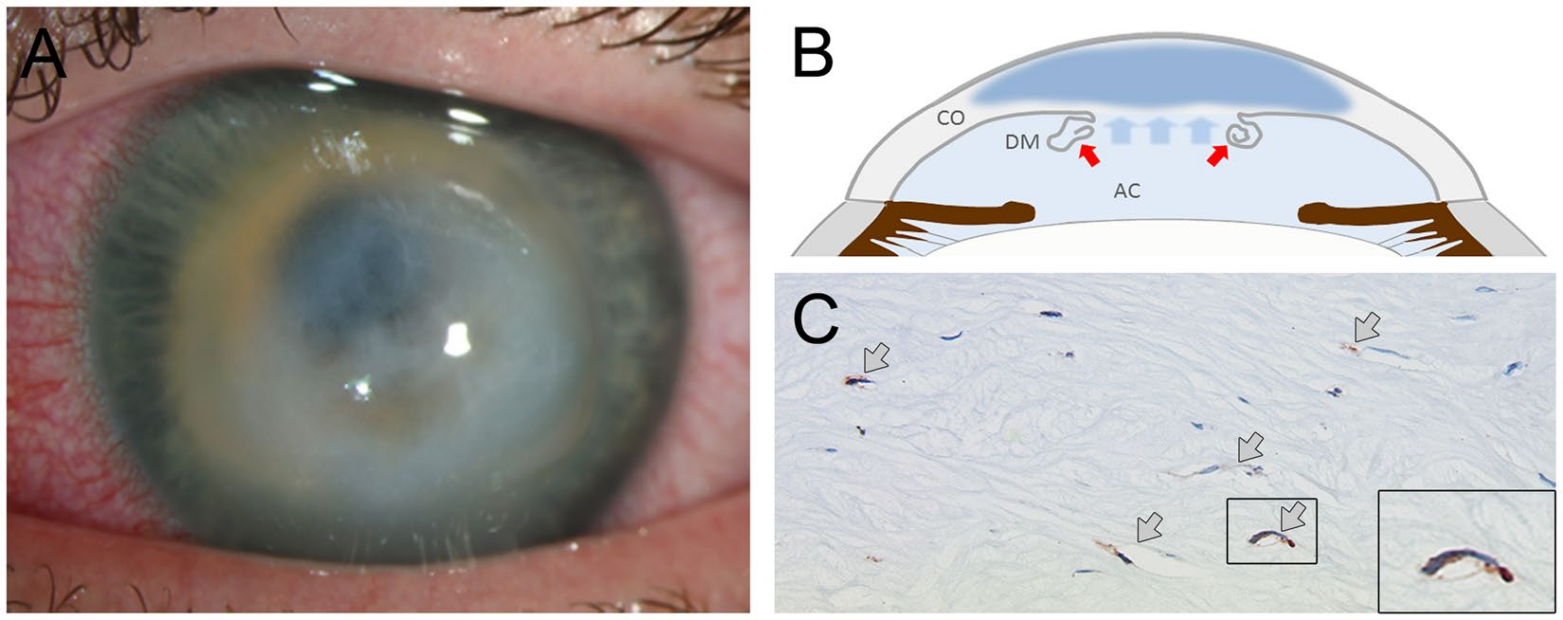
Evidence of isolated corneal lymphangiogenesis in a patient with acute keratoconus. **(A)** Representative clinical image of a patient with keratoconus and acute corneal edema due to spontaneous rupture of the (Descemet´s) basement membrane and subsequent fluid accumulation (acute keratoconus). **(B)** Schematic drawing depicting acute keratokonus: due to rupture of the Descemet´s membrane (DM; red arrows), fluid from the anterior chamber (AC) rapidly accumulates in the cornea (CO), leading to loss of corneal transparency. **(C)** Histological analysis of the cornea from a patient with acute keratoconus obtained after corneal transplantation shows LYVE-1 positive lymphatic vessels in the corneal stroma (arrows) without evidence for blood vessels.

As our animal experiments indicate that corneal lymphangiogenesis is involved in the regulation of at least acute corneal edema, we sought to determine whether lymphatic vessels are also detectable in patients with acute corneal edema (Fig. 3A). For this purpose, we analyzed the cornea of a patient with advanced keratoconus who had developed acute corneal edema due to a spontaneous rupture of the corneal basement membrane (Descemet´s membrane) and subsequent fluid accumulation (Fig. 3A,B). The patient’s cornea was obtained after corneal transplantation, which was performed 2 weeks after Descemet´s membrane rupture, and was evaluated for the presence of lymphatic vessels. Notably, LYVE-1 positive lymphatic vessels were clearly detectable in the corneal stroma (Fig. 3C). In contrast, blood vessels were not detectable. Thus, our results demonstrate that also in the human setting, acute corneal edema is accompanied by the selective ingrowth of corneal lymphatic vessels.

## Discussion

A variety of studies demonstrate that pathological lymphatic vessels are critical for the development of graft rejection after corneal transplantation, dry eye disease, or ocular allergy, and targeting corneal lymphangiogenesis has emerged as a promising approach to treat these diseases^5, 17–19, 22–25^. However, we questioned whether lymphatic vessels at the cornea might also have beneficial functions, as in several extraocular tissues it was shown that lymphatic vessels mediate physiological wound repair and regulate edema^1, 26^. Recently, we could demonstrate that lymphatic vessels support the egress of macrophages form the inflamed cornea, facilitating the resolution of inflammation^31^. In the current study, we additionally analyzed whether lymphatic vessels are also involved in the physiological corneal wound healing response using a mouse model of perforating incision injury, which is known to induce transient corneal edema and opacification^30^. Importantly, our study indicates that in this injury model, selective growth of corneal lymphatic vessels (without concurrent blood vessel growth) seems to play a role in the regulation of corneal edema and the reestablishment of corneal transparency. Consistently, specific blockade of corneal lymphangiogenesis results in a slight but significant delay in the resolution of corneal edema and possibly prolonged corneal opacification. This is in line with previously observed findings after experimental corneal transplantation, where blockade of corneal lymphangiogenesis results in a slight delay of corneal clearing (unpublished, DH, FB, CC), although long-term graft survival is improved^17, 22, 32^.

Although the differences in CCT between the anti-VEGFR-3 treated corneas and controls reached statistical significance 3 weeks after injury, analysis of earlier time points showed no difference in CCT. Furthermore, corneal opacification was also similar between anti-VEGFR-3 treated eyes and controls. There might be several possible explanations for the rather small impact of VEGFR-3 blockade on the resolution of corneal edema and opacification. Although treatment with anti-VEGFR-3 significantly reduced corneal lymphangiogenesis after injury, inhibition was not complete. Therefore, it is possible that the remaining lymphatic vessels were still capable of draining large portions of corneal edema. Furthermore, it is also conceivable that other factors are involved in the resolution process. In this context, it has previously been shown that lymphatic drainage is also regulated by other vascular endothelium-specific factors^33^, the Angiopoetin-Tie system^34, 35^ or by several inflammatory chemo-/cytokines including CCL21^36^, TNF-α^37^, or IL-7^38^. The carbonic anhydrase activity and pump function of the corneal endothelium is also capable to drain large amounts of intracorneal fluid and might also be responsible for the marginal impact of VEGFR-3 blockade on corneal edema resolution after incision injury.

Lymphatic vessels were also detectable in the cornea of a patient who had developed acute corneal swelling due spontaneous rupture of the Descemet´s membrane in acute keratoconus. This indicates that corneal lymphangiogenesis might be involved in the regulation of at least acute corneal edema also in the clinical setting. However, we could analyze only one patient with acute corneal edema, as human histological specimens with acute corneal edema (without concurrent inflammation) are extremely rare. This is because corneal transplantation is usually performed at much later time points (e.g. one year) after Descemet´s membrane rupture, when corneal edema has declined and an inert corneal scar has developed to ensure better suturing and stability of the graft. Nevertheless, further analyses of patient samples with acute corneal edema are necessary to verify our pilot results.

In summary, we demonstrate that a perforating corneal incision injury transiently and selectively induces corneal lymphangiogenesis. These lymphatic vessels seem to contribute to the regulation of acute corneal edema and transparency. To our knowledge, this is the first report that a primarily avascular site is transiently invaded by lymphatic vessels during the physiological healing course. Our results might lead to novel treatment strategies for several blinding eye diseases due to excessive corneal edema and swelling, such as acute keratoconus or corneal endothelial dystrophies. In fact, these diseases are the most common indications for corneal transplantation and are among the most common causes for blindness worldwide^39–43^.

## Methods

### Animals

Animal protocols were approved by the local animal care committee (Landesamt fuer Natur, Umwelt und Verbraucherschutz Nordrhein-Westfalen; AZ 2015.A487) and were in accordance with the Association for Research in Vision and Ophthalmology´s Statement for the Use of Animals in Ophthalmology and Vision Research. Mice were 8 to 12 weeks old female C57BL/6 mice. For corneal incision injury, mice were anaesthetized with an intraperitoneal injection of a combination of ketamine (100 mg/kg) and xylazine hydrochloride (10 mg/kg).

### Corneal incision injury

A central perforating corneal incision injury was generated in the right eye of C57BL/6 mice as previously described^30^. 20 minutes before injury, one eye drop of atropine sulfate (1%, Ursapharm GmbH, Saarbrücken, Germany) was applied to avoid iris incarceration^30^. Afterwards, the central cornea was gently marked with a trephine (1.0 mm diameter) and a linear perforating incision was performed with a surgical blade (Fig. 1A,B). Mice received ofloxacin eye drops after injury (Floxal EDO, Dr. Mann Pharma GmbH, Berlin, Germany) twice daily for the first 3 days and were monitored daily during the first week and then weekly until the end of the experiment. In indicated experiments, mice received the anti-VEGFR3 antibody mF4-31C1 (Eli Lilly and Company, New York, NY, USA; 500 µg, intraperitoneally) directly before, on day 3, 6, 9, and 12 after surgery or equal amounts of PBS.

### Analysis of corneal hem- and lymphangiogenesis after corneal incision injury

For the assessment of corneal hem- and lymphangiogenesis, corneas (n = 5–10 per analyzed time point) were excised at indicated time points after incision injury, fixed in acetone and stained with a rabbit anti-mouse LYVE-1 antibody (AngioBio, Del Mar, CA) and a rat anti-mouse CD31 antibody (Acris Antibodies, Herford, Germany). Whole mount pictures were assembled automatically from 9 to 12 pictures taken at 100x magnification. Afterwards, the area covered with blood and lymphatic vessels was detected with a semiautomatic algorithm established in the image analyzing program CellF (Soft Imaging System) as previously described^44^. Briefly, before analysis, gray value images of the whole mount pictures were modified using several filters, vessels were then detected by threshold setting including the bright vessels and excluding the dark background.

### mRNA isolation and real-time PCR

Central corneas without the limbus were excised before, 1 week, 2 weeks and 4 weeks after incision injury and RNA was isolated using RNeasy Micro Kit (Qiagen, Valencia, CA). Complementary DNA (cDNA) was synthesized with random hexamers using reverse transcriptase (SuperScript III; Invitrogen, Carlsbad, CA). Primers (MWG Biotech, Ebersberg, Germany) were designed using Primer3 software and BLAST (Basic Local Alignment Search Tool, NCBI). PCR reactions (25 μl) contained 20 ng of cDNA, 0.4 µM of each forward and reverse primer, and master mix (SsoFast EvaGreen Supermix; Bio-Rad, Hercules, CA). Real-time PCR was performed under the following conditions: 95 °C for 2 minutes, 40 cycles at 95 °C for 5 seconds and 60 °C (for 15 seconds, followed by 95 °C for 60 seconds and a subsequent melt curve analysis to check amplification specificity. Real-time PCR results were analyzed by the comparative threshold cycle method with HPRT as the endogenous reference gene. The relative mRNA levels of uninjured corneas were chosen as the normalized controls for all analyzed time points after injury. All assays were performed in triplicate and a non-template control was included in all experiments to exclude DNA contamination. Primer sequences and product sizes are summarized in the Supplemental Table 1.

### Assessment of central corneal thickness

Central corneal thickness (CCT) as a measure of corneal edema was analyzed in *in vivo* optical coherence tomography (OCT) scans of the anterior segment. OCT scans were obtained using an 840 nm Spectral Domain OCT device specifically developed for investigating mouse eyes (OCT-HSM, OptoMedical Technologies GmbH, Luebeck, Germany). The OCT device performs 21.000 A-Scans per second, delivering an axial scan resolution about 5.7μm in tissue. Volume scans of the central cornea were obtained after thorough adjustment ensuring a rectangular incidence of the light to avoid refractive aberrations. B-Scans parallel adjacent to the corneal incision were then analyzed and CCT was calculated as the mean value of corneal thickness within the central region of interest measured with 15 pixels lateral width (accords to approximately 75 µm on the corneal surface) using an open source image processing software (ImageJ, NIH, Bethesda, Maryland, USA). CCT was calculated by using intensity depth plots to determine corneal boundaries and under consideration of the refractive index of corneal tissue.

### Assessment of corneal opacity

For the analysis of corneal opacity, slit-lamp photographs were taken every week after injury. To grade corneal opacity, following scoring system, which is commonly used in the assessment of corneal transplants, was used: 0: clear cornea; 1: minimal, superficial (non-stromal) opacity, pupil margin and iris vessels readily visible; 2: minimal, deep (stromal) opacity, pupil margins and iris vessels visible; 3: moderate stromal opacity, only pupil margin visible; 4: intense stromal opacity, only a portion of pupil margin visible; 5: maximum stromal opacity, anterior chamber not visible.

### Immunohistological analysis of a cornea obtained from a patient with acute edema in keratoconus

A male patient (age 26 years) with advanced keratoconus developed acute corneal edema due a rupture of the corneal basement membrane (Descemet´s membrane) and subsequent fluid accumulation. 2 weeks later, penetrating keratoplasty was performed and the patient’s own cornea was immunohistologically analyzed for the presence of lymphatic vessels. For this purpose, the cornea was fixed in phosphate-buffered saline (PBS) containing 4% formaldehyde, rinsed in PBS, dehydrated via graded alcohol series and embedded in paraffin. Serial cross sections (3 µm) were prepared and stained with an antibody directed against LYVE-1 (rabbit anti-human, Zytomed, Berlin, Germany). Negative control staining was performed by omission of the primary antibody and resulted in no staining. Conjunctiva was taken as positive control and revealed specific staining.

Histological experiments were performed in accordance with all relevant guidelines and regulations and were approved by the institutional committee (Ethikkommission der Medizinischen Fakultaet der Universitaet zu Koeln). Informed consent was obtained from the patient.

### Statistical Analyses

Statistical analyses were performed with Microsoft Excel (Microsoft Corp, Redmond, WA) and InStat Version 3.06 (GraphPad Software Inc., San Diego, CA). Statistical significance was determined using the student’s t-test for parametric and the Mann-Whitney-U test for non-parametric variables. P-values < 0.05 were considered statistically significant. Graphs were drawn using Prism4 version 4.03 (GraphPad Software Inc., San Diego, CA). All data are reported as the mean + standard deviation (SD) unless otherwise indicated.

### Data availability statement

The datasets generated during and analyzed in this study are available from the corresponding author on reasonable request.

## Electronic supplementary material

Supplementary Dataset 1
