# Supplementary material for: Transient Ingrowth of Lymphatic Vessels into the Physiologically Avascular Cornea Regulates Corneal Edema and Transparency
Source: Sci Rep. 2017 Aug 3;7:7227. doi: 10.1038/s41598-017-07806-4 (PMC5543160; doi:10.1038/s41598-017-07806-4)
Supplement: Supplementary file 1 — Supplementary Dataset 1 [file 41598_2017_7806_MOESM1_ESM.doc]

**Transient Ingrowth of Lymphatic Vessels into the Physiologically Avascular Cornea Regulates Corneal Edema and Transparency**

Deniz Hos^1,2^,*, Anne Bukowiecki^1^, Jens Horstmann^1,3^, Felix Bock^1,2^, Franziska Bucher^1^, Ludwig M. Heindl^1^, Sebastian Siebelmann^1^, Philipp Steven^1,3^, Reza Dana^4^, Sabine A. Eming^2,3,5^, Claus Cursiefen^1,2^

^1^ Department of Ophthalmology, University of Cologne, Germany

^2^ Center for Molecular Medicine Cologne (CMMC), University of Cologne, Germany

^3^ Excellence Cluster: Cellular Stress Responses in Aging-associated Diseases, CECAD, University of Cologne, Germany

^4^ Schepens Eye Research Institute, Boston, MA, USA

^5^ Department of Dermatology, University of Cologne, Germany

*Correspondence: deniz.hos@uk-koeln.de

**Supplemental figures:**


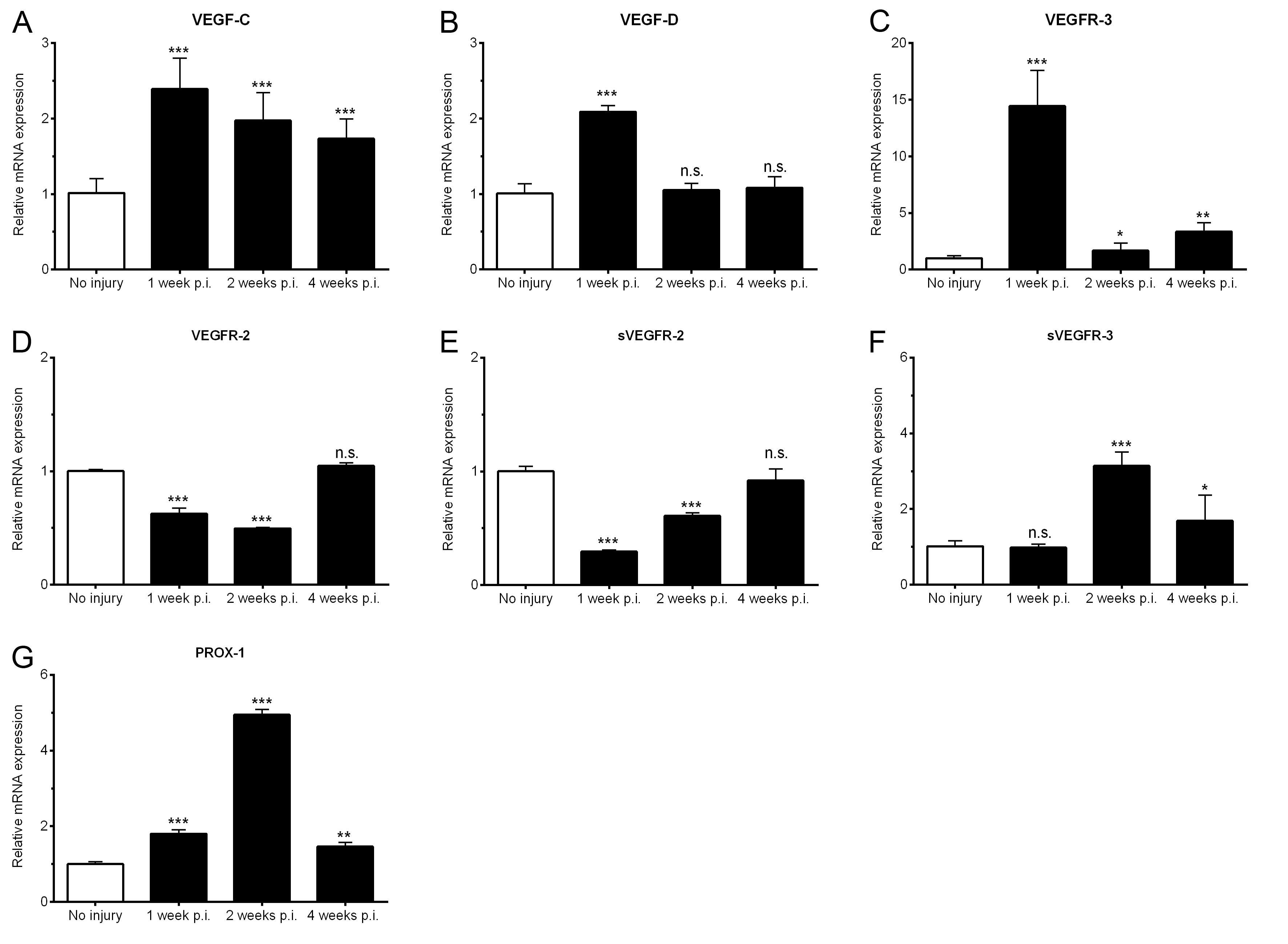


**Supplemental Figure 1. Changes in corneal gene expression after incision injury.** Real-time PCR demonstrating changes of VEGF-C **(A)**, VEGF-D **(B),** VEGFR-3 **(C)**, VEGFR-2 **(D)**, soluble VEGFR-2 **(E)**, soluble VEGFR-3 **(F)**, and PROX-1 **(G)** mRNA expression in the cornea after incision injury. Data are shown as mean + SD. Note the difference in y-axis scaling. p.i.: post injury; *: p<0.05; **: p<0.01; ***: p<0.001; n.s.: not significant.

**
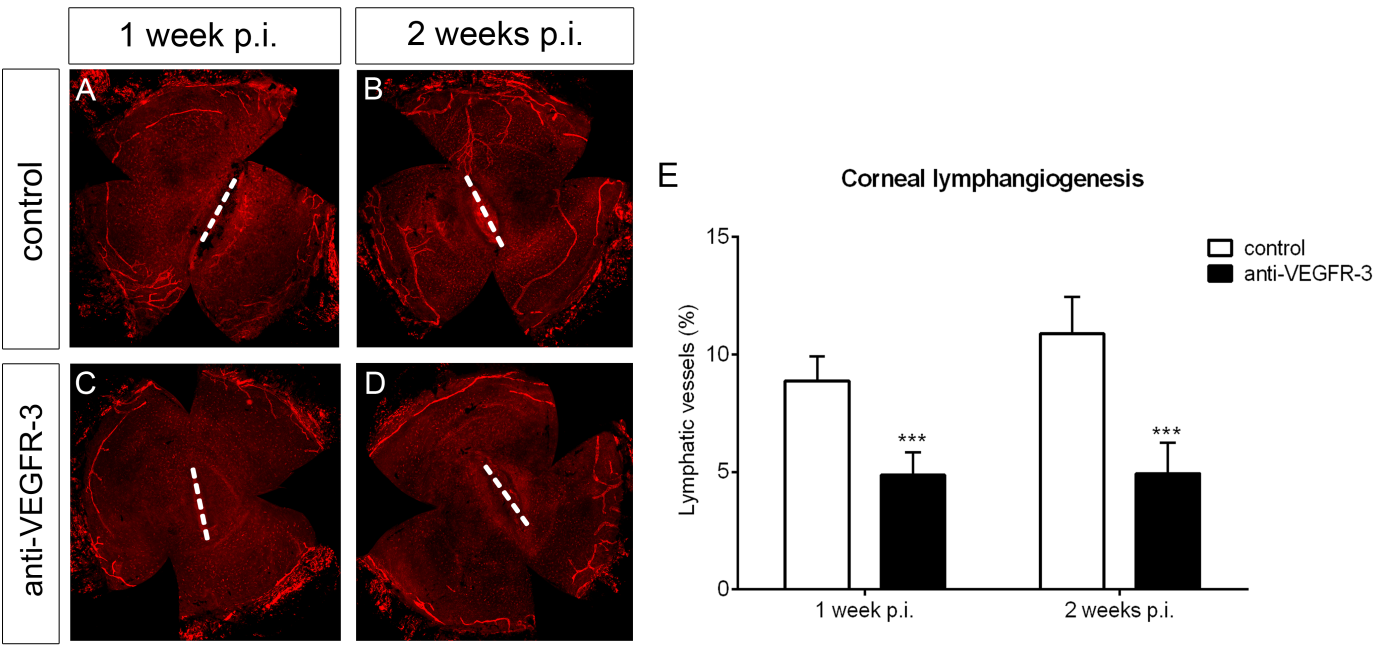
**

**Supplemental Figure 2. Treatment with the anti-VEGFR-3 antibody mF4-31C1** **blocks corneal lymphangiogenesis after incision injury**. Mice were treated with the anti-VEGFR-3 antibody mF4-31C1 (500 µg, intraperitoneally) directly before, on day 3, 6, 9, and 12 after incision injury or equal amounts of PBS; **(A to D)** Corneal whole mounts stained with LYVE-1 (red); dashed lines: area of incision injury; p.i.: post injury. **(E)** Quantification of corneal lymphangiogenesis; data are shown as mean + SD; *: p<0.05; n.s.: not significant.

**Supplemental table:**

| **Gene** | **Product size (bp)** | **Sequence (5'–3')** |
| --- | --- | --- |
| HPRT | 163 | for: GTTGGATACAGGCCAGACTTTGTTG  rev: GATTCAACTTGCGCTCATCTTAGGC |
| VEGF-C | 219 | for: AGAACGTGTCCAAGAAATCAGC  rev: ATGTGGCCTTTTCCAATACG |
| VEGF-D | 86 | for: ATGGCGGCTAGGTGATTCC  rev: CCCTTCCTTTCTGAGTGCTG |
| VEGFR-3 | 94 | for: GTCCCTCTACTTCCAACTGCTTC  rev: CACTCCTCCTCTGTGACTTTGAG |
| VEGFR-2 | 128 | for: ATTCTGGACTCTCCCTGCCTAC  rev: GCTCTTTCGCTTACTGTTCTGG |
| sVEGFR-2 | 218 | for: CACCAGTTTGCAAGAACTTGGATGCT  rev: AATTCTGTCACCCAGGGATGC |
| sVEGFR-3 | 89 | for: CTGTGAACCCCATCGAGAGT  rev: GCTTCAGCTTGTCTGGACCT |
| PROX-1 | 177 | for: CAGGAGATGGCTGAGAACAAG  rev: AGAGGGTTTGGCTGAGAAGAC |

**Supplemental Table 1. Primer used for real-time PCR.**
